# A Systematic Summary of Systematic Reviews on Anticoagulant Therapy in Sepsis

**DOI:** 10.3390/jcm8111869

**Published:** 2019-11-04

**Authors:** Shuhei Murao, Kazuma Yamakawa

**Affiliations:** Division of Trauma and Surgical Critical Care, Osaka General Medical Center, Osaka 558-8558, Japan; shmu20268271@gmail.com

**Keywords:** sepsis, disseminated intravascular coagulation, coagulopathy, anticoagulant, antithrombin, thrombomodulin, heparin, recombinant activated protein C, umbrella review, systematic review

## Abstract

Many systematic reviews have been published regarding anticoagulant therapy in sepsis, among which there is substantial heterogeneity. This study aimed to provide an overview of existing systematic reviews of randomized controlled trials by using a comprehensive search method. We searched MEDLINE, EMBASE, and Cochrane Database of Systematic Reviews. Of 895 records screened, 19 systematic reviews were included. The target agent was as follows: antithrombin (*n* = 4), recombinant thrombomodulin (*n* = 3), heparin (*n* = 3), recombinant activated protein C (*n* = 8), and all anticoagulants (*n* = 1). Antithrombin did not improve mortality in critically ill patients but indicated a beneficial effect in sepsis-induced disseminated intravascular coagulation (DIC), although the certainty of evidence was judged as low. Recombinant thrombomodulin was associated with a trend in reduced mortality in sepsis with coagulopathy with no increased risk of bleeding, although the difference was not statistically significant and the required information size for any declarative judgement insufficient. Although three systematic reviews showed potential survival benefits of unfractionated heparin and low-molecular-weight heparin in patients with sepsis, trials with low risk of bias were lacking, and the overall impact remains unclear. None of the meta-analyses of recombinant activated protein C showed beneficial effects in sepsis. In summary, a beneficial effect was not observed in overall sepsis in poorly characterized patient groups but was observed in sepsis-induced DIC or sepsis with coagulopathy in more specific patient groups. This umbrella review of anticoagulant therapy suggests that characteristics of the target populations resulted in heterogeneity among the systematic reviews.

## 1. Introduction

Sepsis is a life-threatening condition with high morbidity and mortality that remains an important public health problem. Systemic activation of the coagulation system is frequently observed in patients with sepsis, and mortality increases in accordance with the increased severity of the coagulopathy [1,2,3]. Several anticoagulants agents, such as antithrombin, recombinant activated protein C (rAPC), recombinant thrombomodulin (rTM), heparin, and tissue factor pathway inhibitor, have been expected and evaluated as adjunctive therapy for the management of sepsis [4,5,6]. The increasing number of clinical trials on the topic of anticoagulants has created a need to organize the data and summarize the generated evidence.

A systematic review is helpful in summarizing the current evidence for a particular clinical question and in serving as the basis for clinical practice guidelines [7,8]. Many systematic reviews have been published pertaining to anticoagulants therapy [9,10,11]. However, there is substantial heterogeneity among these systematic reviews, and clinical evidence for the therapy remains controversial.

We conducted an umbrella review of existing systematic reviews and meta-analyses of randomized controlled trials of anticoagulant therapy selected via a comprehensive search method to provide an overview of its efficacy and safety and to explore the causes of heterogeneity among the systematic reviews.

## 2. Method

Study reporting is provided in accordance with the Preferred Reporting Items for Systematic Reviews and Meta-Analyses (PRISMA) statement. Our umbrella review followed the methodology published elsewhere [12]. We developed a protocol before conducting the analysis and registered it in the PROSPERO database (registration no. CRD 42019134671).

### 2.1. Search Strategy

We searched MEDLINE (source, PubMed), EMBASE, and the Cochrane Database of Systematic Reviews for articles pertaining to anticoagulant therapy in patients with sepsis. The search strategy was restricted to systematic reviews and meta-analyses of randomized controlled trials published before 23 May 2019. Non-English-language articles were excluded. The search was performed using the combinations of the three groups of search terms shown below. The final search terms and results are shown in the Supplementary Material (Table S1).

1. Anticoagulants OR Heparin OR LMWH OR UFH OR Danaparoid OR Protease Inhibitors OR Gabexate OR Nafamostat OR Thrombomodulin OR Tissue-factor-pathway Inhibitor OR Antithrombin OR Protein C.
2. Sepsis OR Systemic Inflammatory Response Syndrome OR Hemorrhagic Disorders OR Blood Coagulation Disorders OR Thrombophilia OR Disseminated Intravascular Coagulation.
3. Systematic Review OR Meta-analysis.

### 2.2. Study Selection and Inclusion Criteria

Citations were stored and duplicates were excluded using EndNote software. Two independent reviewers (K.Y. and S.M.) screened the titles and abstracts of the studies and subsequently reviewed the full-text articles. The reviewers resolved disagreements through discussion and consensus. If necessary, authors of the included articles were contacted to clarify any unclear information. We included studies with the following characteristics:

1. Study types: Systematic reviews and meta-analyses of randomized controlled trials.
2. Population: Sepsis patients regardless of the causes and age.
3. Intervention: Any anticoagulants such as antithrombin, rTM, unfractionated heparin and low-molecular weight heparin, recombinant human activated protein C, and tissue pathway factor inhibitor.
4. Control: No restriction on controls.
5. Outcomes: All-cause mortality and bleeding complications.

### 2.3. Data Extraction

Two independent reviewers (K.Y. and S.M.) extracted the data using a standardized data extraction form, with disagreements resolved by discussion and consensus. We identified the following information for each trial: lead author’s name, year of publication, inclusion criteria, number of patients and trials, type of anticoagulants, and outcome measures. All-cause mortality and bleeding complications were investigated as outcome measures. The definition of bleeding complications was followed as proposed by the authors of the individual studies.

### 2.4. Quality Assessment and Data Synthesis

The quality of the included systematic reviews was assessed using the Assessing the Methodological Quality of Systematic Reviews (AMSTAR) instrument, which is a measurement tool to assess the quality of systematic reviews. Two authors provided a narrative and quantitative synthesis of the included studies, which were structured around the type of intervention, characteristics of the target population and the outcomes, with discrepancies resolved by discussion.

## 3. Results

### 3.1. Literature Search

**Figure 1:**
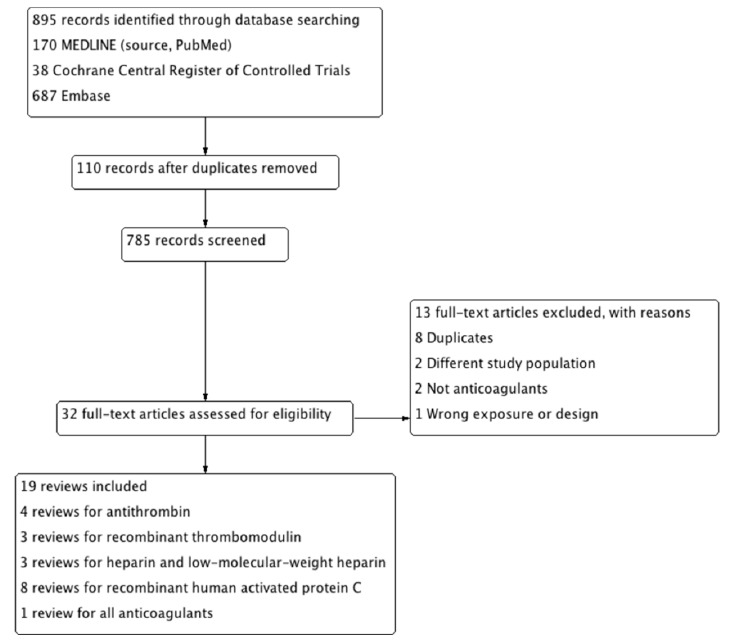
Preferred Reporting Items for Systematic Reviews and Meta-Analyses (PRISMA) chart for identification and selection of studies for inclusion.

Figure 1 shows the PRISMA flow chart of study selection in the umbrella review. We identified 19 systematic reviews of randomized controlled trials.

### 3.2. Included Studies

Study characteristics are summarized in Table 1. The target agent of 19 systematic reviews is as follows: antithrombin (*n* = 4) [10,11,12,13], rTM (*n* = 3) [9,14,15], heparin and low-molecular-weight heparin (LMWH) (*n* = 3) [16,17,18], recombinant human activated protein C (*n* = 8) [19,20,21,22,23,24,25,26], and all anticoagulants (*n* = 1), which included all anticoagulant agents and evaluated separate meta-analyses in different populations [27].

## 4. Summary of Systematic Reviews

**Figure 2:**
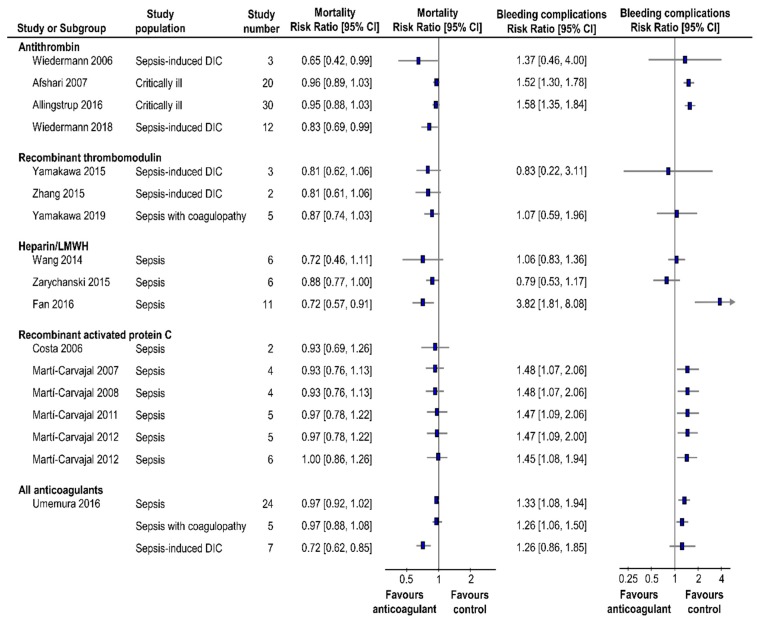
Summary of the findings of mortality and bleeding complications. CI = confidence interval; DIC = disseminated intravascular coagulation; LMWH = low-molecular-weight heparin.

Figure 2 summarizes the data on mortality and bleeding complications of the included systematic reviews.

### 4.1. Antithrombin

We found four published systematic reviews of antithrombin [10,11,13,28], among which we observed significant heterogeneity. The reason for this heterogeneity depended on the results of the KyperSept trial, a large-scale, multicenter, randomized controlled trial, which failed to show an improvement in mortality in patients with severe sepsis [4].

Two reviews focused on its effect in critically ill patients and showed no statistically significant effect on mortality [10,28]. Antithrombin significantly increased bleeding complications. Trial sequential analysis showed that there was sufficient evidence to reject a beneficial effect of more than 10% relative risk reduction on mortality. Based on this result, the Survival Sepsis Campaign (SSC) guidelines 2016 recommended against the use of antithrombin in sepsis and septic shock patients.

In contrast, two systematic reviews focused on patients with sepsis-induced disseminated intravascular coagulation (DIC) and reported beneficial effects on mortality [11,13]. In this meta-analysis, post hoc analyses of the KyperSept trial accounted for 51% relative weight. This sub-analysis evaluated the antithrombin effect in KyperSept patients with DIC who did not receive concomitant heparin, and antithrombin-treated patients were associated with reduced mortality (28-day mortality: 22.2% with antithrombin vs. 40.0% with placebo, *p* < 0.01) [29]. Because a post hoc study contains high risk of bias, this meta-analysis concluded that the quality of evidence supporting the use of antithrombin in sepsis-induced DIC was low. On the basis of similar results, the Japanese Clinical Practice Guidelines for Management of Sepsis and Septic shock weakly recommended the use of antithrombin for DIC patients with reduced antithrombin activities [30].

### 4.2. Recombinant Thrombomodulin

We identified three systematic reviews of randomized controlled trials [9,14,15]. Two similar systematic reviews were published in 2015 and 2016 [14,15]. They reported that rhTM was associated with a trend in reduced mortality, but statistical significance was not reached. Considering these results, the SSC guidelines 2016 mentioned its beneficial effect for the first time. However, it refrained from recommending the use of rhTM because the multicenter phase III randomized controlled trial (Sepsis Coagulopathy Asahi Recombinant LE Thrombomodulin (SCARLET) study) was ongoing at that time.

In August 2018, a press release for the SCARLET trial was published [31], which reported that no statistically significant difference was observed in 28-day all-cause mortality, the difference being 2.6%. Following this result, an updated systematic review was conducted to assess the efficacy and safety of rhTM for the treatment of sepsis with coagulopathy [9]. An approximately 13% reduction in mortality was observed, but the difference was not significant (RR = 0.87, 95% CI = 0.74–1.03, *I*^2^ = 0%). rhTM did not increase the risk of serious bleeding complications. Trial sequential analysis indicated that because only 42% of the required information size was fulfilled at this stage, no declarative judgement could be made, and further trials were warranted.

### 4.3. Unfractionnated Heparin and Low-Molucular-Weight Heparin

Of the three included systematic reviews, two evaluated the efficacy of unfractionated heparin and LMWH and one included only LMWH [16,17,18]. In most of the included studies, unfractionated heparin and LMWH were administered for prophylactic purposes and dosed for deep vein thrombosis. Heparin administration was associated with reduced mortality without increasing bleeding complications, although trials with low risk of bias were lacking [16,17]. Fan et al. evaluated only for LMWH, which significantly reduced mortality but increased bleeding complications in sepsis. Because of limited data in the English literature, only trials published in Chinese were included in this study.

Following these results, the SSC guidelines 2016 reported potential benefits of unfractionated heparin and LMWH. However, it suspended its recommendation until further randomized controlled trials are conducted.

### 4.4. Recombinant Activated Protein C

We found nine systematic reviews of rAPC, and seven meta-analyses were available [18,19,20,21,22,23,24,25]. All meta-analyses reported the consistent conclusion that rAPC did not show a beneficial effect in sepsis patients, but it increased the risk of bleeding.

rAPC was originally recommended in the SSC guidelines 2004 and 2008 after the Recombinant Human Protein C Worldwide Evaluation in Severe Sepsis (PROWESS) trial, a multinational, multicenter, phase III, randomized controlled trial, showed significantly improved mortality in patients with sepsis (28-day mortality: 24.7% with rAPC vs. 30.8% with placebo, *p* = 0.005) [32]. However, subsequent clinical trials have failed to show an improvement in mortality [33]. Finally, rAPC was not shown to be effective for sepsis and septic shock by the Prospective Recombinant Human Activated Protein C Worldwide Evaluation in Severe Sepsis and Septic Shock (PROWESS-SHOCK) trial and was withdrawn from the market [6].

### 4.5. All Anticoagulant Therapy Classified to Specific Population

Umemura et al. conducted a separate meta-analysis of randomized controlled trials for all anticoagulant therapy in three specific populations: sepsis, sepsis with coagulopathy, and sepsis-induced DIC [27]. There was no significant reduction in mortality in the sepsis and sepsis with coagulopathy populations. However, a significant reduction in mortality was observed in sepsis-induced DIC (RR = 0.72, 95% CI = 0.62–0.85, *I^2^* = 0%; 7 trials with 1603 participants). Bleeding complications consistently showed a similar increasing trend in all three populations. Considering the balance of its risks and benefits, anticoagulant therapy was recommended only for sepsis-induced DIC.

## 5. Discussion

Our umbrella review examined the current evidence from systematic reviews of randomized controlled trials evaluating anticoagulant therapy for sepsis. We identified adequate systematic reviews targeted to overall sepsis patients, most of which did not show improved mortality, except for the prophylactic use of heparin [10,16,17,18,19,20,21,22,23,24,25,26,28]. In these systematic reviews of antithrombin and rAPC on overall populations with sepsis, large-scale, multinational, multicenter, randomized controlled trials were included, but they failed to show an improvement in mortality in patients with sepsis or septic shock [4,6,32,33]. In the last two decades, the field of sepsis has focused on heterogenous, poorly characterized patient groups as well as on anticoagulant therapy, and yet we still have no new therapies to treat these conditions. However, the response to sepsis is so complicated and personal that no single agent can be effective in all septic patients. Precision medicine and personalized medicine are patient-specific approaches that will allow more precise management of individual patients with sepsis [34,35]. This has already begun with a retrospective analysis of a dataset of randomized controlled trials assessing the correlation between host-response biomarkers and clinical outcomes [36].

Some systematic reviews of anticoagulant therapy were conducted targeting more specific characteristics such as sepsis with coagulopathy or sepsis-induced DIC, and several studies showed favorable results. Recent findings suggested that local thrombosis acts as antimicrobial matrices that mediate host protection against pathogens under certain circumstances during sepsis, which is specifically called “immunothrombosis” [37]. In contrast, the continuous and excessive activation of inflammation could result in the uncontrolled activation of thrombosis [38,39]. Therefore, it is conceivable that anticoagulant therapy would be useful only for septic patients with an excessive coagulation disorder. However, there are insufficient randomized controlled trials designed to target such specific populations at this time, and further well-designed trials are still required.

## 6. Conclusions

In conclusion, a beneficial effect was not observed in overall sepsis in poorly characterized patient groups but was observed in sepsis-induced DIC or sepsis with coagulopathy in more specific patient groups. This umbrella review of anticoagulant therapy suggests that characteristics of the target populations resulted in heterogeneity among the systematic reviews.

## Figures and Tables

**Table 1:** Characteristics of the included systematic reviews.

Type of Anticoagulant	Author	Population	Number of Trials	Number of Patients	AMSTAR
Antithrombin	Wiedermann, 2006 [13]	Sepsis-induced DIC	3	364	10
	Afshari, 2008 [28]	Critically ill	20	3458	11
	Allingstrup, 2016 [10]	Critically ill	30	3933	11
	Wiedermann, 2018 [11]	Sepsis-induced DIC	12	766	7
Thrombomodulin	Yamakawa, 2015 [14]	Sepsis-induced DIC	3	838	10
	Zhang, 2016 [15]	Sepsis-induced DIC	2	821	9
	Yamakawa, 2019 [9]	Sepsis with coagulopathy	5	1762	10
Heparin/	Wang, 2014 [16]	Sepsis	6	604	9
	Zarychanski, 2015 [17]	Sepsis	6	2477	9
Low-molecular-weight heparin	Fan, 2016 [18]	Sepsis	11	594	8
Activated protein C	Kylat, 2006 [19]	Neonate with sepsis	0	NA	11
	Costa, 2007 [20]	Severe sepsis	2	4330	7
	Martí-Carvajal, 2007 [21]	Severe sepsis	4	4911	11
	Martí-Carvajal, 2008 [22]	Severe sepsis	4	4911	11
	Martí-Carvajal, 2011 [24]	Severe sepsis	5	5101	11
	Kylat, 2012 [23]	Neonates with sepsis	0	NA	11
	Martí-Carvajal, 2012 [25]	Severe sepsis	5	5101	11
	Martí-Carvajal, 2012 [26]	Adult and pediatric sepsis	6	6781	11
All anticoagulants	Umemura, 2016 [27]	Sepsis, Sepsis with coagulopathy, Sepsis-induced DIC	24	14767	10

DIC = disseminated intravascular coagulation; NA = not available.

## Supplementary Materials

The following are available online at https://www.mdpi.com/2077-0383/8/11/1869/s1, Table S1: Search terms and results.
