# Supplementary material for: A Systematic Summary of Systematic Reviews on Anticoagulant Therapy in Sepsis
Source: J Clin Med. 2019 Nov 4;8(11):1869. doi: 10.3390/jcm8111869 (PMC6912821; doi:10.3390/jcm8111869)
Supplement: Supplementary file 1 [file jcm-08-01869-s001.pdf]

Table S1. Search terms and results.

Database	Dates searched	Search terms	Results found
MEDLINE (source, PubMed)	1966–May 2019	#1: Anticoagulants[pa] OR Anticoagulant*[tiab]	241815
		#2: Antithrombin[pa] OR Antithrombin[tiab]	15896
		#3: Thrombomodulin[MeSH] OR Thrombomodulin[tiab] OR rhTM[tiab] OR rTM[tiab]	4922
		#4: Protein C[MeSH] OR Protein C[tiab] OR APC[tiab]	38766
		#5: lipoprotein-associated coagulation inhibitor[nm] OR tissue factor pathway inhibitor[nm] OR tissue factor pathway inhibitor[tiab] OR TFPI[tiab]	2554
		#6: Heparin[MeSH] OR Heparin[tiab] OR Heparin, Low-Molecular-Weight[MeSH] OR LMWH [tiab] OR Unfractionated heparin[tiab] OR UFH[tiab]	96081
		#7: Danaparoid[tiab]	417
		#8: Protease inhibitors[pa] OR protease inhibitor*[tiab] OR Gabexate[MeSH] OR Gabexate[tiab] OR Nafamostat[MeSH] OR Nafamostat[tiab]	180973
		#9: #1 OR #2 OR #3 OR #4 OR #5 OR #6 OR #7 OR #8	458319
		#10: Sepsis[MeSH] OR Sepsis[tiab]	169697
		#11: Systemic Inflammatory Response Syndrome[MeSH] OR Systemic Inflammatory Response Syndrome[tiab]	122021
		#12: Multiple organ failure[MeSH] OR multiple organ failure[tiab]	14745
		#13: Shock[MeSH] OR shock[tiab]	201724
		#14: Hemorrhagic Disorders[MeSH] OR Hemorrhagic Disorder*[tiab]	128486
		#15: Blood Coagulation Disorders[MeSH] OR Blood Coagulation Disorder*[tiab]	93821
		#16: Thrombophilia[MeSH] OR Thrombophilia[tiab]	27272
		#17: Disseminated Intravascular Coagulation[MeSH] OR Disseminated Intravascular Coagulation[tiab]	15251
		#18: #11 OR #12 OR #13 OR #14 #15 OR #16 OR #17	53818
		#19: (((systematic review[ti] OR systematic literature review[ti] OR systematic scoping review[ti] OR systematic narrative review[ti] OR systematic qualitative review[ti] OR systematic evidence review[ti] OR systematic quantitative review[ti] OR systematic meta-review[ti] OR systematic critical review[ti] OR systematic mixed studies review[ti] OR systematic mapping review[ti] OR systematic cochrane review[ti] OR systematic search and review[ti] OR systematic integrative review[ti]) NOT comment[pt] NOT (protocol[ti] OR protocols[ti])) NOT MEDLINE [subset]) OR (Cochrane Database Syst Rev[ta] AND review[pt]) OR systematic review[pt]	130973
		#20: #9 AND #18 AND #19	170
Cochrane Central Register of Controlled Trials	May 2019	#1: [mh Anticoagulants] OR (Anticoagulant*):ti,ab,kw	10316
		#2: [mh Antithrombin] OR (antithrombin):ti,ab,kw	2399
		#3: [mh Thrombomodulin] OR (thrombomodulin OR rhTM OR rTM):ti,ab,kw	387

		#4: [mh "Protein C"] OR ("protein C" OR APC):ti,ab,kw	1628
		#5: (lipoprotein-associated coagulation inhibitor*):ti,ab,kw OR (tissue factor pathway inhibitor*):ti,ab,kw OR (TFPI):ti,ab,kw	446
		#6: [mh Heparin] OR [mh "Heparin, Low-Molecular-Weight"] OR (heparin OR "low molecular weight heparin" OR "unfractionated heparin" OR UFH):ti,ab,kw	11455
		#7: (Danaparoid):ti,ab,kw	57
		#8: [mh "Protease inhibitors"] OR [mh Gabexate] OR [mh Nafamostat] OR ((protease NEXT inhibitor*) OR Gabexate OR Nafamostat):ti,ab,kw	8425
		#9: {OR #1-#8}	28448
		#10: [mh Sepsis] OR (sepsis OR Septic):ti,ab,kw	13817
		#11: [mh "Systemic Inflammatory Response Syndrome"] OR ("Systemic Inflammatory Response Syndrome"):ti,ab,kw	4958
		#12: [mh "Multiple organ failure"] OR ("Multiple organ failure"):ti,ab,kw	1343
		#13: [mh Shock] OR (shock):ti,ab,kw	9925
		#14: [mh "Hemorrhagic Disorders"] OR (hemorrhagic NEXT disorder*):ti,ab,kw	2551
		#15: [mh "Blood Coagulation Disorders"] OR ("Blood Coagulation" NEXT Disorder*):ti,ab,kw	1502
		#16: [mh Thrombophilia] OR (thrombophilia):ti,ab,kw	647
		#17: [mh "Disseminated Intravascular Coagulation"] OR ("Disseminated Intravascular Coagulation"):ti,ab,kw	349
		#18: {OR #10-#17}	24789
		#19: #9 and #18 in Cochrane Reviews	29
		#20: (("systematic review" OR "systematic literature review" OR "systematic scoping review" OR "systematic narrative review" OR "systematic qualitative review" OR "systematic evidence review" OR "systematic quantitative review" OR "systematic meta-review" OR "systematic critical review" OR "systematic mixed studies review" OR "systematic mapping review" OR "systematic cochrane review" OR "systematic search and review" OR "systematic integrative review"):ti) NOT ((protocol OR protocols):ti)	4795
		#21: (systematic review):pt	58
		#22: #20 or #21	4824
		#23: #9 and #18 and #22	9
		#24: #9 and #18 and #22 in Cochrane Reviews, Cochrane Protocols	0
		#25: #9 and #18 and #22 in Trials	9
Embase	1974–May 2019	#1: ((EMB.EXACT.EXPLODE("anticoagulant agent")) OR TI,AB(Anticoagulant*))	692426
		#2: ((EMB.EXACT.EXPLODE("antithrombin") OR TI,AB(antithrombin*)))	24729
		#3: ((EMB.EXACT.EXPLODE("thrombomodulin") OR TI,AB(thrombomodulin* OR rhTM OR rTM)))	8397
		#4: ((EMB.EXACT.EXPLODE("protein C") OR TI,AB("protein C" OR APC)))	58702

	#5: ((EMB.EXACT.EXPLODE("tissue factor pathway inhibitor")) OR (TI,AB(("tissue factor pathway" P/2 inhibitor*) OR ("lipoprotein-associated coagulation" P/2 inhibitor*) OR TFPI)))	4876
	#6: ((EMB.EXACT.EXPLODE(heparin OR "low molecular weight heparin")) OR (TI,AB(heparin OR "low-molecular-weight heparin" OR "Unfractionated heparin" OR UFH)))	210383
	#7: TI,AB(danaparoid)	640
	#8: ((EMB.EXACT.EXPLODE("proteinase inhibitor" OR "gabexate" OR "nafamstat")) OR (TI,AB((protease OR proteinase) P/2 inhibitor*) OR gabexate OR nafamstat OR nafamostat))	376064
	#9: (#1 or #2 or #3 or #4 or #5 or #6 or #7 or #8)	1042802
	#10: ((EMB.EXACT.EXPLODE("sepsis") OR (TI,AB(sepsis OR septic))))	317660
	#11: ((EMB.EXACT.EXPLODE("systemic inflammatory response syndrome")) OR (TI,AB("systemic inflammatory response syndrome")))	276634
	#12: ((EMB.EXACT.EXPLODE("multiple organ failure")) OR (TI,AB("multiple organ failure")))	40341
	#13: (((EMB.EXACT.EXPLODE("shock")) OR TI,AB(shock)))	286050
	#14: ((EMB.EXACT.EXPLODE("bleeding disorder")) OR (TI,AB((hemorrhagic OR bleeding) P/2 disorder*)))	17696
	#15: ((EMB.EXACT.EXPLODE("thrombophilia") OR TI,AB(thrombophilia)))	14526
	#16: ((EMB.EXACT.EXPLODE("disseminated intravascular clotting")) OR (TI,AB(disseminated P/2 intravascular P/2 (coagulation OR clotting))))	27467
	#17: (#10 or #11 or #12 or #13 or #14 or #15 or #16)	613344
	#18: (TI("systematic review" OR "systematic literature review" OR "systematic scoping review" OR "systematic narrative review" OR "systematic qualitative review" OR "systematic evidence review" OR "systematic quantitative review" OR "systematic meta-review" OR "systematic critical review" OR "systematic mixed studies review" OR "systematic mapping review" OR "systematic cochrane review" OR "systematic search and review" OR "systematic integrative review"))	123365
	#19: ((EMB.EXACT.EXPLODE("systematic review") OR (JN("Systematic Reviews"))) AND DTYPE(review))	114011
	#20: ((#18 OR #19) NOT TI(protocol OR protocols))	183973
	#21: (#9 and #17 and #20)	687
